# Assessment of Medication Adherence Among Heart Failure Patients in an Ambulatory Care Setting: A Prospective Observational Study

**DOI:** 10.3390/jcm15135230

**Published:** 2026-07-04

**Authors:** Morouj Bakr, Loay Milibari, Sara El Khansa, Ebtisam Alkhattaby, Sumaia Jambi, Ibrahim Jelaidan, Rana Almuwallad, Amjad Madkhali, Ashjan Altuwrqi, Mawaddah Alrhily, Sarah Alharbi, Lama Alfehaid, Sanaa AlSulami

**Affiliations:** 1 Department of Pharmaceutical Care Services, King Abdulaziz Medical City, Riyadh 11426, Saudi Arabia; 2 King Abdullah International Medical Research Center, Jeddah 21423, Saudi Arabia; 3 College of Medicine, King Saud Bin Abdulaziz University for Health Sciences, Jeddah 21423, Saudi Arabia; 4 Department of Pharmaceutical Care Services, King Abdulaziz Medical City, Jeddah 21423, Saudi Arabia; 5 Nursing Services, King Abdulaziz Medical City, Jeddah 21423, Saudi Arabia; 6 Department of Cardiology, King Abdulaziz Medical City, Jeddah 21423, Saudi Arabia; 7 College of Pharmacy, Umm Al-Qura University, Makkah 24381, Saudi Arabia; 8 College of Pharmacy, King Abdulaziz University, Jeddah 21589, Saudi Arabia; 9 King Abdullah International Medical Research Center, Riyadh 11481, Saudi Arabia; 10 Department of Pharmacy Practice, College of Pharmacy, King Saud Bin Abdulaziz University for Health Sciences, Riyadh 14611, Saudi Arabia

**Keywords:** heart failure, adherence, discrepancy, health literacy

## Abstract

**Background:** Medication non-adherence remains a major challenge among patients with heart failure (HF) and is associated with adverse clinical outcomes, including increased hospitalization and mortality. Prescription discrepancies may further contribute to poor adherence and unfavorable outcomes. This study aimed to assess the prevalence of medication non-adherence and prescription discrepancies, identify associated factors, and evaluate their relationship with HF-related rehospitalization. **Methods:** This prospective observational study included adult patients with HF attending an ambulatory HF clinic between July 2023 and April 2024. The primary outcomes were the proportion of patients demonstrating medication adherence and the prevalence, number, and types of prescription discrepancies. Secondary outcomes included assessment of health literacy, identification of factors associated with medication non-adherence, and evaluation of HF-related rehospitalization and its association with medication non-adherence, prescription discrepancies, and health literacy. **Results:** A total of 202 patients were included. The mean age was 60.4 ± 14.1 years, and 69.3% were male. High medication adherence was observed in 43.6% of patients, while 39.6% and 16.8% demonstrated medium and low adherence, respectively. Prescription discrepancies were identified in 104 patients (51.5%), resulting in 178 discrepancy events, most of which were patient-generated (70.2%). Marginal and inadequate health literacy were identified in 51.5% and 24.8% of patients, respectively. Polypharmacy, limited health literacy, younger age, and adverse medication effects were significantly associated with medication non-adherence. During the six-month follow-up, 18 patients (8.9%) experienced HF-related rehospitalization. Medium/low medication adherence, prescription discrepancies, and limited health literacy were independently associated with rehospitalization in multivariable logistic regression analysis. **Conclusions:** Medication non-adherence and prescription discrepancies were highly prevalent among HF patients in the ambulatory care setting and were significantly associated with HF-related rehospitalization. Limited health literacy and polypharmacy represented major contributors to non-adherence. These findings underscore the need for multidisciplinary interventions, medication reconciliation, and patient-centered educational strategies to improve medication management and clinical outcomes in HF patients.

## 1. Introduction

Heart failure (HF) is a complex clinical syndrome characterized by the inability of the heart to sufficiently pump blood to meet the metabolic needs of the body. It is a growing public health problem in Saudi Arabia, where the incidence of HF is increasing. This is attributed to various factors such as an aging population, high prevalence of risk factors like diabetes and hypertension, and lifestyle changes [1].

Medication non-adherence is a critical problem among patients with HF and can be associated with increased morbidity, mortality, and healthcare costs [2]. The World Health Organization has identified adherence to long-term therapies as a major determinant of treatment effectiveness and health outcomes in chronic diseases, emphasizing that improving adherence may have a greater impact on population health than advances in specific medical treatments [3]. Medication non-adherence is defined as the extent to which a patient’s medication-taking behavior differs from the agreed-upon plan of their healthcare provider [4].

Studies among HF patients in the U.S. showed that non-adherent patients have a mortality rate that is 1.5 to 2 times higher than adherent patients, and a hospitalization rate that is up to 4 times higher [5,6]. Additionally, existing studies have shown that medication non-adherence is prevalent among cardiac patients in Saudi Arabia and has adverse impacts on patients’ health outcomes and healthcare resources [7,8,9]. A prospective study was conducted to measure medication adherence using the Medication Event Monitoring System (MEMS) to predict rehospitalization and mortality. The results suggest that non-adherence contributes to higher hospitalization rates [10]. Another cross-sectional study was conducted in tertiary care centers in Riyadh to determine the prevalence of medication adherence at different levels among cardiac patients and to assess whether age is correlated with medication adherence. It was found that 33.7% of participants had low adherence levels. Moreover, there was a positive correlation between age and adherence. The authors of this study emphasized the need for further research to identify the causes and potential solutions for this issue among cardiac patients [7].

Measuring medication adherence can be challenging; however, several validated tools and scores have been developed to assess it, including MEMS, pill counts, pharmacy refill records, and self-reported measures such as the Morisky Medication Adherence Scale (MMAS-8) [11,12,13].

Prescription discrepancy is a significant factor that can affect medication non-adherence among HF patients [4]. It is defined as any difference in medications, doses, or frequencies between the patient’s medication list and their electronic medical records, leading to confusion for the patient [14,15]. Examples of patient-related discrepancies (PRD) include failure to take prescribed medications or deviations from prescribed doses and frequencies, whereas healthcare-system-related discrepancies (HSRD) may include omissions or inaccuracies in the electronic medical record. Such discrepancies can contribute to medication confusion, non-adherence, and adverse clinical outcomes [15,16].

Despite previous studies, there remains a gap in understanding the factors contributing to medication non-adherence among HF patients in Saudi Arabia, including the roles of polypharmacy, health literacy, adverse medication effects, and access to healthcare [17].

Accordingly, this study aimed to assess medication adherence and prescription discrepancies among patients with heart failure attending an ambulatory clinic, identify factors associated with non-adherence, including health literacy, and evaluate their association with heart failure-related rehospitalization.

## 2. Materials and Methods

### 2.1. Study Design and Participants

This prospective observational study was conducted at the ambulatory HF clinic at King Abdulaziz Medical City, Ministry of National Guard Health Affairs, Jeddah, Saudi Arabia, an urban tertiary-care academic medical center that provides specialized cardiovascular services.

Patients who were 18 years old or older with confirmed diagnoses of HF and on guideline-directed medical therapy (GDMT) who were attending our HF ambulatory clinic between July 2023 and April 2024 were included. Patients who received follow-up exclusively through the HF telehealth clinic or had incomplete medication adherence or health literacy assessments were excluded from the study.

### 2.2. Data Collection and Follow-Up

Following Institutional Review Board (IRB) approval from the King Abdullah International Medical Research Center (KAIMRC), eligible patients with HF attending the ambulatory HF clinic were evaluated by a HF specialist. During each clinic visit, the specialist assessed the patient’s clinical status, reviewed HF medications, and identified medication discrepancies, medication non-adherence, and associated factors. This information was documented in the patients’ electronic medical records. Subsequently, a pharmacy resident reviewed the clinical documentation and collected study variables, including demographic characteristics, medication-related factors, and prescription discrepancies. All data were securely recorded in a password-protected data collection sheet to maintain confidentiality.

Each patient was interviewed by an HF nurse specialist using two validated questionnaires. Medication adherence was assessed using the validated Arabic version of the 8-item MMAS-8, a widely used self-reported instrument that evaluates medication-taking behavior across multiple dimensions. MMAS-8 scores were categorized according to established criteria as high adherence (score = 8), medium adherence (score 6 to <8), and low adherence (score <6) [13]. These three categories were used for descriptive reporting of medication adherence. For comparative and regression analyses, patients with medium and low adherence were combined into a single non-adherent group, whereas patients with high adherence were classified as adherent. This approach was adopted to facilitate clinically meaningful comparisons and improve statistical power, particularly given the relatively small number of patients in the low-adherence category.

Health literacy was assessed using the validated Arabic version of the Three-item Brief Health Literacy Screen (BHLS), a brief screening tool developed to identify patients with limited health literacy [18]. The BHLS includes three questions evaluating the frequency of needing assistance with reading hospital materials, the difficulty in learning about medical conditions from written information, and confidence in independently completing medical forms. Higher scores indicate better health literacy.

Polypharmacy was defined as the concurrent use of five or more chronic medications [19]. Limited access to healthcare was defined as documented or patient-reported difficulty obtaining medications, attending scheduled clinic appointments, or accessing healthcare services because of transportation, scheduling, medication availability, or other logistical barriers.

Prescription discrepancies were categorized according to the primary source contributing to the discrepancy. PRD included missed medications, self-adjusted doses or frequencies, medication discontinuation without provider recommendation, and failure to initiate prescribed therapies. HSRD included prescribing errors, omissions, inaccurate medication documentation, or discrepancies within the electronic medical record. Mixed discrepancies were classified when both patient-related and healthcare-system-related factors contributed to the discrepancy.

After the initial clinic visit, all patients were followed for 6 months to capture HF-related emergency department visits and hospitalizations. Associations between rehospitalization and medication adherence, prescription discrepancies, and health literacy were subsequently evaluated.

### 2.3. Endpoints

The primary endpoints were the proportion of HF patients demonstrating medication adherence in the ambulatory care setting and the prevalence, number, and types of medication discrepancies. Secondary endpoints included assessment of health literacy levels and their association with medication non-adherence, identification of factors associated with medication non-adherence, and evaluation of HF-related rehospitalization and its association with medication non-adherence, prescription discrepancies, and limited health literacy.

### 2.4. Statistical Analysis

Descriptive statistics were used to summarize baseline characteristics and study outcomes. Categorical variables were reported as frequencies and percentages, while continuous variables were presented as mean ± standard deviation (SD). Comparative analyses between adherent and non-adherent groups were performed using the chi-square test or Fisher’s exact test for categorical variables and the independent t-test for continuous variables, as appropriate. Variables associated with heart failure rehospitalization were further evaluated using univariable and multivariable logistic regression analyses, and the results were reported as odds ratios (ORs) or adjusted odds ratios (aORs) with 95% confidence intervals (CIs). All statistical analyses were performed using Stata Statistical Software, version 19.0 (StataCorp LLC, College Station, TX, USA). A two-sided *p*-value <0.05 was considered statistically significant.

## 3. Results

### 3.1. Study Population

**Figure 1:**
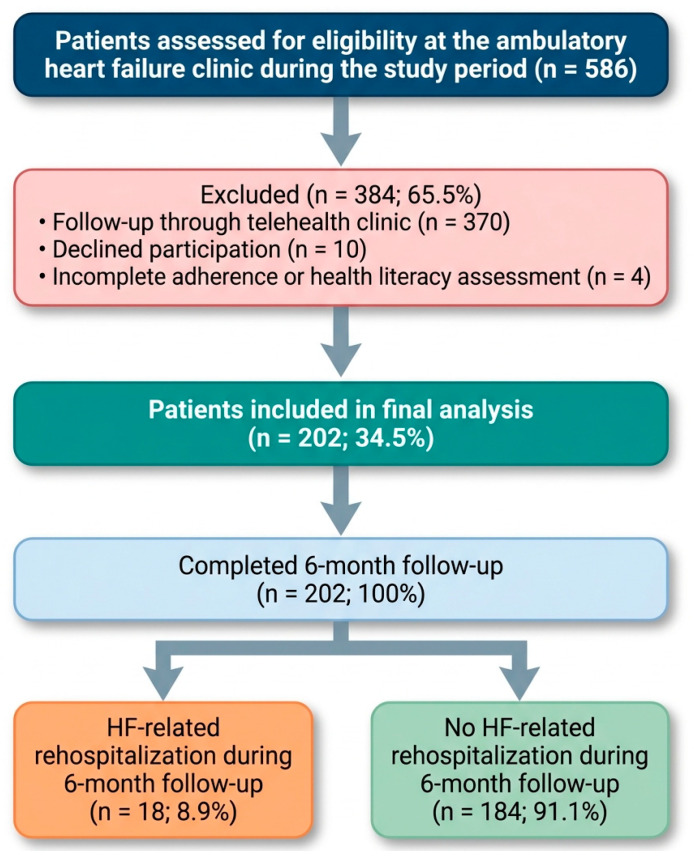
Patient flow diagram.

The study flow is presented in Figure 1. A total of 586 patients attending the ambulatory HF clinic were assessed during the study period. Of these, 384 patients were excluded, primarily because follow-up was conducted through telehealth clinics (*n* = 370), while 10 patients declined participation and 4 had incomplete medication adherence or health literacy assessments. The remaining 202 patients met the eligibility criteria, completed study assessments, and were included in the final analysis. All included patients completed the six-month follow-up period. During follow-up, 18 patients (8.9%) experienced HF-related rehospitalization, whereas 184 patients (91.1%) did not.

A total of 586 patients were screened for eligibility. After exclusion of 384 patients, 202 patients met the eligibility criteria and were included in the final analysis. All included patients completed the 6-month follow-up period, during which 18 patients (8.9%) experienced HF-related rehospitalization.

### 3.2. Baseline Characteristics

A total of 202 patients with HF were included in the study. Baseline characteristics for the overall cohort and stratified by medication adherence status are presented in Table 1. The mean age was 60.4 ± 14.1 years, and the majority of patients were male (69.3%). The mean body weight was 80.5 ± 18.8 kg. Non-adherent patients had a significantly lower mean age than adherent patients (58.0 ± 15.6 vs. 63.5 ± 11.2 years, *p* = 0.006).

HF with reduced ejection fraction (HFrEF) was the predominant phenotype (80.7%), followed by HF with mildly reduced ejection fraction (HFmrEF; 13.8%) and HF with preserved ejection fraction (HFpEF; 5.4%). The mean left ventricular ejection fraction (LVEF) was 32.5 ± 9.9%. Patients experienced a mean of 0.78 ± 1.39 HF-related hospitalizations during the preceding 12 months.

Baseline characteristics were generally comparable between adherent and non-adherent groups.

### 3.3. Baseline Guideline-Directed Medical Therapy

Baseline use of guideline-directed medical therapy (GDMT) is presented in Table 2. All patients were receiving beta-blockers (100%). Other commonly prescribed therapies included sodium–glucose cotransporter-2 inhibitors (SGLT2i) (93.1%), mineralocorticoid receptor antagonists (MRA) (77.2%), and loop diuretics (74.3%). Among renin–angiotensin–aldosterone system (RAAS) inhibitors, angiotensin receptor–neprilysin inhibitors (ARNI) were the most frequently used (63.4%), followed by angiotensin-converting enzyme inhibitors (15.3%) and angiotensin receptor blockers (7.9%). Less frequently used medications included digoxin (14.4%), isosorbide dinitrate (16.8%), hydralazine (10.9%), and ivabradine (5.9%).

### 3.4. Primary Outcomes

#### 3.4.1. Medication Adherence

Medication adherence was assessed using the MMAS-8 scale. Overall, 88 patients (43.6%) demonstrated high adherence, while 80 (39.6%) and 34 (16.8%) exhibited medium and low adherence, respectively. These findings indicate substantial variability in adherence levels within the study population.

#### 3.4.2. Medication Discrepancies

A total of 178 medication discrepancies were identified among 104 patients, representing 51.5% of the study population. Because some patients experienced more than one discrepancy, analyses were performed according to the total number of discrepancy events. These discrepancies were categorized as PRD, HSRD, or mixed discrepancies.

PRD were the most common, accounting for 70.2% (*n* = 125) of all discrepancies. These primarily included deviations from prescribed dosing or frequency and failure to take prescribed medications. Loop diuretics were the medication class most frequently associated with dose or frequency discrepancies (*n* = 76), although similar discrepancies were also observed with other medication classes at lower frequencies. SGLT2 inhibitors were most frequently associated with failure to take prescribed medications (*n* = 49).

HSRD accounted for 8.4% (*n* = 15) and were mainly related to omissions in the electronic medical record (EMR), particularly involving SGLT2 inhibitors.

**Figure 2:**
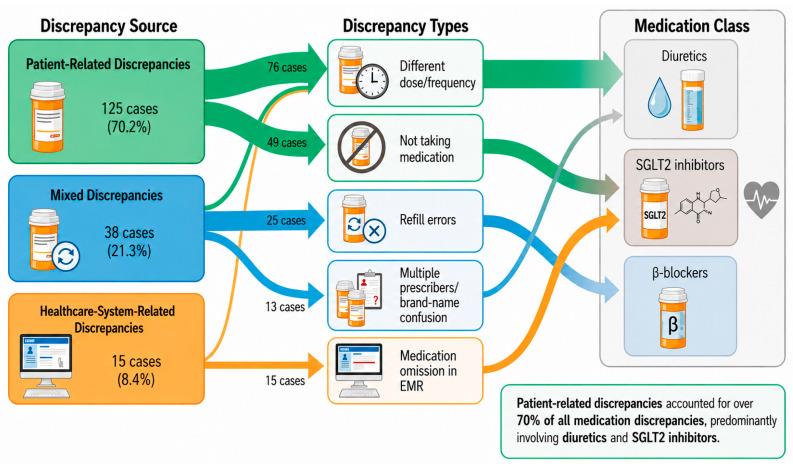
Distribution of medication discrepancies according to discrepancy source, discrepancy type, and associated medication classes among heart failure patients.

Mixed discrepancies represented 21.3% of cases. These were largely driven by refill-related errors (*n* = 25), predominantly involving beta-blockers, as well as discrepancies related to multiple prescribers or different medication brand names (*n* = 13) (Figure 2).

The figure highlights the predominant medication class involved in each discrepancy type and does not imply that discrepancies occurred exclusively within that medication class.

### 3.5. Secondary Outcomes

#### 3.5.1. Levels of Health Literacy

As part of the secondary outcomes, health literacy level was assessed using the BHLS. The results revealed that nearly half of the study population (51.5%) had marginal health literacy. An additional 23.8% of patients had adequate health literacy, while 24.8% had inadequate health literacy.

#### 3.5.2. Descriptive Factors Associated with Medication Non-Adherence

**Figure 3:**
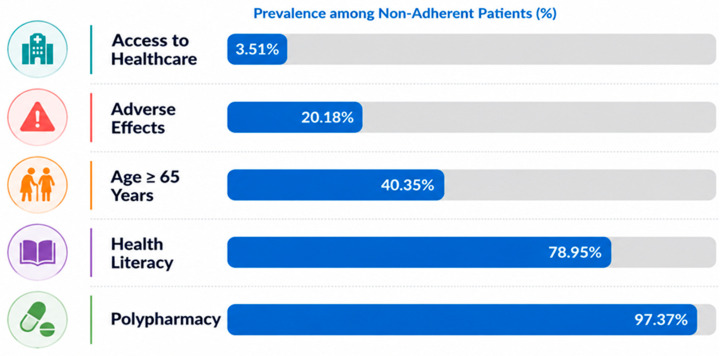
Prevalence of factors associated with medication non-adherence among heart failure patients, highlighting polypharmacy and limited health literacy as the most common contributors.

Multiple factors associated with medication non-adherence were identified among the study population (Figure 3). Polypharmacy was the most common factor, affecting 97.4% of non-adherent patients. Marginal or inadequate health literacy was also highly prevalent, affecting 78.9% of non-adherent patients. Older age (≥65 years) and adverse medication effects were observed in 40.4% and 20.2% of non-adherent patients, respectively. Limited access to healthcare was the least common factor, reported in 3.5% of non-adherent patients.

#### 3.5.3. Inferential Analysis of Factors Associated with Medication Non-Adherence and HF Rehospitalization

Inferential analysis demonstrated that non-adherent patients were significantly younger and more likely to experience polypharmacy, marginal or inadequate health literacy, and adverse medication effects compared with adherent patients (Table 3). No significant differences were observed regarding sex, body weight, LVEF, HF phenotype, or prior HF hospitalizations.

Exploratory multivariable logistic regression analysis demonstrated that medium/low medication adherence was independently associated with HF-related rehospitalization (adjusted odds ratio [aOR] 5.60, 95% confidence interval [CI] 1.22–25.75; *p* = 0.023). Prescription discrepancies (aOR 4.18, 95% CI 1.11–15.71; *p* = 0.034) and marginal/inadequate health literacy (aOR 3.02, 95% CI 1.01–9.03; *p* = 0.047) were also independently associated with rehospitalization.

#### 3.5.4. Rate of Heart Failure Rehospitalization

At the six-month follow-up, 18 patients (8.9%) experienced HF-related rehospitalization. When stratified by medication adherence status, HF-related rehospitalization occurred in 13 of 114 patients (11.4%) with medium/low adherence compared with 5 of 88 patients (5.7%) with high adherence.

Patients who experienced HF-related rehospitalization were more likely to have medium/low medication adherence (72.2% vs. 54.9%), prescription discrepancies (100% vs. 46.7%), and marginal or inadequate health literacy (88.9% vs. 65.2%) compared with patients who were not rehospitalized (Table 4). Polypharmacy was present in all rehospitalized patients and in 90.8% of non-rehospitalized patients, while age ≥ 65 years was observed in 50.0% and 41.3% of patients, respectively.

In univariable analyses, marginal/inadequate health literacy was significantly associated with HF-related rehospitalization (OR 4.26, 95% CI 1.20–15.16), whereas medium/low medication adherence demonstrated a numerical association (OR 2.14, 95% CI 0.78–5.87). Exploratory multivariable logistic regression analysis demonstrated that medium/low medication adherence was independently associated with HF-related rehospitalization (aOR 5.60, 95% CI 1.22–25.75; *p* = 0.023). Prescription discrepancies (aOR 4.18, 95% CI 1.11–15.71; *p* = 0.034) and marginal/inadequate health literacy (aOR 3.02, 95% CI 1.01–9.03; *p* = 0.047) were also independently associated with HF-related rehospitalization. Polypharmacy (aOR 1.88, 95% CI 0.42–8.45; *p* = 0.41) and age ≥ 65 years (aOR 1.29, 95% CI 0.51–3.24; *p* = 0.58) were not independently associated with rehospitalization.

## 4. Discussion

Our results demonstrate significant challenges with medication adherence and suggest key considerations for optimizing HF management. The study found that 43.6% (*n* = 88) of patients had high medication adherence, 39.6% (*n* = 80) medium, and 16.8% (*n* = 34) low. Our findings were in line with previous studies in Saudi Arabia, including the Aseer region, which reported poor medication adherence among patients with HF and other cardiovascular conditions [9,20]. This high prevalence of non-adherence is concerning, as poor medication adherence has consistently been associated with increased morbidity, mortality, and healthcare costs among patients with chronic diseases and HF [3,5,6,20].

The high prevalence of medication discrepancies (51.5%) suggests significant deficits in medication management. This rate is higher than that reported in a study conducted at King Fahad Medical City, Riyadh, which identified medication discrepancies in 23.2% of cardiac patients [21]. In addition, 178 discrepancy events were identified in 104 patients, indicating that multiple discrepancy events may have occurred in some patients. The multifactorial nature of these discrepancies, involving patient- and healthcare system-related factors, underscores the complexity of medication management in HF patients and the need for comprehensive strategies to address these issues [14,15,16].

Interestingly, non-adherent patients had a significantly lower mean age than adherent patients (58.0 ± 15.6 vs. 63.5 ± 11.2 years; *p* = 0.006). Although older age has traditionally been considered a risk factor for medication non-adherence because of factors such as polypharmacy, cognitive impairment, and treatment complexity [22], our findings suggest that younger patients with HF may also represent a vulnerable subgroup. This observation may reflect competing work and family responsibilities, lower perceived disease severity, or differences in health behaviors among younger patients. Therefore, adherence-promoting interventions should not be limited to older adults but should also consider the unique barriers faced by younger patients with HF. In addition, male patients were numerically more likely to be non-adherent to medication, consistent with findings from the United Arab Emirates [23]. However, sex differences were not statistically significant in our cohort.

The distribution of HF phenotypes was similar among the adherent and non-adherent groups. HFrEF was the predominant phenotype in both groups. This suggests that HF phenotype itself may not significantly affect medication adherence.

The health literacy assessment showed that 51.5% of patients had marginal health literacy and 24.8% had inadequate health literacy. Limited health literacy was significantly more common among non-adherent patients and was an independently associated factor of HF rehospitalization. These findings are clinically relevant, as low health literacy has been associated with poorer health outcomes and increased healthcare utilization [24,25]. Furthermore, the prevalence of limited health literacy observed in our study is consistent with findings from a recent study of patients with acute coronary syndrome and heart failure in Qatar [24]. Limited health literacy was more common in our cohort than previously reported in a systematic review of patients with HF [26], underscoring the need for patient-centered educational interventions tailored to patients with limited health literacy.

Polypharmacy was very common among non-adherent patients and was one of the strongest factors associated with medication non-adherence. This finding is consistent with previous literature demonstrating poorer adherence among patients with increasing medication burden and multimorbidity [19,27]. In addition, adverse medication effects were significantly more frequent in non-adherent patients, highlighting the importance of individualized medication review and optimization.

PRD accounted for 70.2% of all discrepancy events, indicating the importance of patient-related factors in medication management problems. The most common mismatches were incorrect dosing or frequency of loop diuretics and failure to take prescribed SGLT2 inhibitors. These findings are clinically relevant because these medications are central to guideline-directed HF management and may influence HF stability and clinical outcome. Additional mixed discrepancies, such as refill-related errors and confusion regarding multiple prescribers or medication brand names, highlight the need for improved medication reconciliation processes and communication between healthcare providers and patients [14,15,16].

HSRD accounted for a smaller proportion of discrepancy events (8.4%), but omissions in the electronic medical record involving SGLT2 inhibitors remained clinically important. These findings suggest that opportunities remain to improve the accuracy of medication documentation and continuity of care despite advances in electronic prescribing systems [15,16].

An additional clinically important finding of this study is the observed association between medication adherence, prescription discrepancies, health literacy, and HF-related rehospitalization. During the 6-month follow-up period, HF-related rehospitalization occurred in 18 patients (8.9%). When stratified by adherence status, rehospitalization occurred in 13 of 114 patients (11.4%) with medium/low adherence compared with 5 of 88 patients (5.7%) with high adherence, suggesting a clinically meaningful relationship between lower medication adherence and rehospitalization risk. Rehospitalized patients also demonstrated higher rates of prescription discrepancies and marginal or inadequate health literacy. Exploratory multivariable logistic regression analysis demonstrated that medium/low medication adherence, prescription discrepancies, and limited health literacy were independently associated with HF-related rehospitalization.

These findings should be interpreted cautiously, given the relatively small number of rehospitalization events, the observational nature of the study, and the potential for residual confounding. Furthermore, socioeconomic variables such as educational attainment, income level, and employment status were not collected and therefore could not be evaluated. Although all participants received care within a healthcare system that provides outpatient services and prescribed medications without direct patient charges, unmeasured socioeconomic factors may still influence medication adherence through mechanisms such as health literacy, social support, and health-related behaviors. Nevertheless, the results underscore the clinical importance of optimizing medication management, improving health literacy, and addressing medication discrepancies as potential targets for reducing rehospitalizations and improving patient outcomes. These findings further support the growing emphasis on comprehensive ambulatory HF management strategies that integrate medication optimization, patient education, medication reconciliation, and close outpatient follow-up to prevent disease progression and recurrent hospitalization [28].

## 5. Conclusions

Medication non-adherence and medication discrepancies are still very common in patients with HF and are linked to adverse clinical outcomes, including HF-related rehospitalization. More than half of patients experienced at least one prescription discrepancy, and over half demonstrated medium or low medication adherence. PRD accounted for the largest proportion of identified medication discrepancies. The most common factors associated with non-adherence were polypharmacy, limited health literacy, and adverse medication effects. These findings underscore the need for multidisciplinary interventions, medication reconciliation, patient education, and optimization of healthcare system processes to improve adherence and decrease the clinical burden of heart failure.

## Figures and Tables

**Table 1:** Baseline Characteristics of the Study Population Overall and Stratified by Medication Adherence Status at Baseline.

Parameter	Adherent * (*n* = 88)	Non-Adherent ** (*n* = 114)	*p*-Value
Age (years), mean ± SD	63.5 ± 11.2	58.0 ± 15.6	**0.006**
Gender, *n* (%)			0.54
Male	59 (67.0)	81 (71.0)	
Female	29 (32.9)	33 (28.9)	
Weight (kg), mean ± SD	78.8 ± 19.3	81.9 ± 18.4	0.24
Types of HF, *n* (%)			0.94
HFrEF	70 (79.5)	93 (81.5)	
HFmrEF	13 (14.7)	15 (13.1)	
HFpEF	5 (5.6)	6 (5.2)	
LVEF %, mean ± SD	32.8 ± 9.3	32.3 ± 10.3	0.72
Number of HF hospitalizations in past 12 months, mean ± SD	0.8 ± 1.6	0.7 ± 1.2	0.63

Data are presented as *n* (%) unless otherwise indicated. Continuous variables were compared using an independent t-test, while categorical variables were compared using a chi-square test or Fisher’s exact test, as appropriate. A *p*-value < 0.05 was considered statistically significant. HF: heart failure; HFrEF: heart failure with reduced ejection fraction; HFmrEF: heart failure with mildly reduced ejection fraction; HFpEF: heart failure with preserved ejection fraction; LVEF: left ventricular ejection fraction; SD: standard deviation. * Adherent: MMAS-8 score = 8. ** Non-adherent: MMAS-8 score < 8 (combined medium- and low-adherence categories).

**Table 2:** Baseline Guideline-Directed Medical Therapy (GDMT).

Baseline Medications	Total (*n* = 202)	Adherent * (*n* = 88)	Non-Adherent ** (*n* = 114)	*p*-Value
Beta-blockers	202 (100)	88 (100)	114 (100)	—
Digoxin	29 (14.4)	17 (19.3)	12 (10.5)	0.09
Ivabradine	12 (5.9)	4 (4.5)	8 (7.0)	0.47
RAAS inhibitors				
ACEi	31 (15.3)	10 (11.3)	21 (18.4)	0.17
ARB	16 (7.9)	10 (11.3)	6 (5.3)	0.11
ARNI	128 (63.4)	57 (64.7)	71 (62.2)	0.71
Diuretics				
Loop diuretics *	150 (74.3)	68 (77.3)	82 (71.9)	0.39
Metolazone	3 (1.5)	1 (1.1)	2 (1.7)	0.74
MRA	156 (77.2)	70 (79.5)	86 (75.4)	0.49
Isosorbide dinitrate	34 (16.8)	12 (13.6)	22 (19.3)	0.29
Hydralazine	22 (10.9)	10 (11.3)	12 (10.5)	0.85
SGLT2 inhibitors	188 (93.1)	83 (94.3)	105 (92.1)	0.54

Data are presented as *n* (%). Categorical variables were compared using the chi-square test or Fisher’s exact test, as appropriate. *p*-values were not calculated for variables with no between-group variability. Loop diuretics included furosemide and bumetanide. ACEi: angiotensin-converting enzyme inhibitor; ARB: angiotensin receptor blocker; ARNI: angiotensin receptor neprilysin inhibitor; MRA: mineralocorticoid receptor antagonist; SGLT2i: sodium–glucose cotransporter-2 inhibitor. * Adherent: MMAS-8 score = 8. ** Non-adherent: MMAS-8 score < 8 (combined medium- and low-adherence categories).

**Table 3:** Inferential Analysis of Factors Associated with Medication Non-Adherence Among Heart Failure Patients.

Variable	Adherent (*n* = 88)	Non-Adherent (*n* = 114)	*p*-Value
Age (years), mean ± SD	63.5 ± 11.2	58.0 ± 15.6	0.006
Male sex, *n* (%)	59 (67.0)	81 (71.0)	0.54
Weight (kg), mean ± SD	78.8 ± 19.3	81.9 ± 18.4	0.24
LVEF (%), mean ± SD	32.8 ± 9.3	32.3 ± 10.3	0.72
HF hospitalization in the prior 12 months, mean ± SD	0.8 ± 1.6	0.7 ± 1.2	0.63
Polypharmacy, *n* (%)	74 (84.1)	111 (97.4)	0.001
Marginal/Inadequate health literacy, *n* (%)	46 (52.3)	90 (78.9)	<0.001
Age ≥ 65 years, *n* (%)	39 (44.3)	46 (40.4)	0.57
Adverse medication effects, *n* (%)	8 (9.1)	23 (20.2)	0.03
Limited access to healthcare, *n* (%)	1 (1.1)	4 (3.5)	0.27

Continuous variables were analyzed using an independent t-test, while categorical variables were analyzed using chi-square test or Fisher’s exact test, as appropriate. A *p*-value < 0.05 was considered statistically significant. HF: heart failure; LVEF: left ventricular ejection fraction; SD: standard deviation.

**Table 4:** Association Between Medication Non-Adherence/Prescription Discrepancies and Heart Failure Rehospitalization.

Variable	Rehospitalized (*n* = 18)	Not Rehospitalized (*n* = 184)	OR (95% CI)	aOR (95% CI)	*p*-Value
Medium/Low medication adherence, *n* (%)	13 (72.2)	101 (54.9)	2.14 (0.78–5.87)	5.60 (1.22–25.75)	0.023
Presence of prescription discrepancy, *n* (%)	18 (100)	86 (46.7)	—	4.18 (1.11–15.71)	0.034
Marginal/Inadequate health literacy, *n* (%)	16 (88.9)	120 (65.2)	4.26 (1.20–15.16)	3.02 (1.01–9.03)	0.047
Polypharmacy, *n* (%)	18 (100)	167 (90.8)	—	1.88 (0.42–8.45)	0.41
Age ≥ 65 years, *n* (%)	9 (50.0)	76 (41.3)	1.42 (0.55–3.66)	1.29 (0.51–3.24)	0.58

Data are presented as *n* (%) unless otherwise indicated. Univariable associations were evaluated using the chi-square test or Fisher’s exact test, as appropriate. Multivariable logistic regression was performed to identify independent risk factors for HF rehospitalization. OR: odds ratio; aOR: adjusted odds ratio; CI: confidence interval. ORs could not be reliably estimated for variables with complete separation.

## Data Availability

All data generated or analyzed during this study are included in this article.

## Abbreviations

ACEi	Angiotensin-Converting Enzyme Inhibitor
aOR	Adjusted Odds Ratio
ARB	Angiotensin Receptor Blocker
ARNI	Angiotensin Receptor Neprilysin Inhibitor
BHLS	Brief Health Literacy Screening Tool
CI	Confidence Interval
EMR	Electronic Medical Record
GDMT	Guideline-Directed Medical Therapy
HF	Heart Failure
HFimpEF	Heart Failure with Improved Ejection Fraction
HFmrEF	Heart Failure with Mildly Reduced Ejection Fraction
HFpEF	Heart Failure with Preserved Ejection Fraction
HFrEF	Heart Failure with Reduced Ejection Fraction
HSRD	Healthcare System-Related Discrepancies
LVEF	Left Ventricular Ejection Fraction
MMAS-8	8-item Morisky Medication Adherence Scale
MRA	Mineralocorticoid Receptor Antagonist
OR	Odds Ratio
PRD	Patient-Related Discrepancies
RAAS	Renin–Angiotensin–Aldosterone System
SD	Standard Deviation
SGLT2i	Sodium–Glucose Cotransporter-2 Inhibitor
SPSS	Statistical Package for the Social Sciences
STATA	Stata Statistical Software
